# Awareness regarding antimicrobial resistance and confidence to prescribe antibiotics in dentistry: a cross-continental student survey

**DOI:** 10.1186/s13756-022-01192-x

**Published:** 2022-12-11

**Authors:** Aya Bajalan, Tiina Bui, Gabriela Salvadori, Dalton Marques, Alison Schumacher, Cassiano Kuchenbecker Rösing, Ulf Reidar Dahle, Fernanda Cristina Petersen, Antônio Pedro Ricomini-Filho, Belinda Farias Nicolau, Roger Junges

**Affiliations:** 1 Institute of Oral Biology, Faculty of Dentistry, University of Oslo, Oslo, Norway; 2 Piracicaba Dental School, University of Campinas, Piracicaba, Brazil; 3 Faculty of Dentistry, University of Toronto, Toronto, ON Canada; 4 Faculty of Dentistry, Federal University of Rio Grande do Sul, Porto Alegre, Brazil; 5 Centre for Antimicrobial Resistance, Norwegian Institute of Public Health, Oslo, Norway; 6 Faculty of Dental Medicine and Oral Health Sciences, McGill University, Montreal, Canada

**Keywords:** Antimicrobial stewardship, Antibiotic resistance, Microbiology, Health education, Cross-sectional survey, Drug prescriptions

## Abstract

**Background:**

The antimicrobial resistance (AMR) crisis is a major global threat and one of its biggest drivers is the overuse of antibiotics in humans. Dentists are responsible for 5–10% antibiotic prescriptions worldwide and recent data suggest that knowledge and prescribing practices need improvement.

**Methods:**

A cross-sectional web-survey was sent to dental students from six universities in Norway, Canada, and Brazil. Topics addressed covered awareness, confidence to prescribe antibiotics, and education needs. Data were presented descriptively and statistical testing was employed to compare group means when applicable.

**Results:**

In total, 562 responses were collected across the three countries with a response rate of 28.6%. ‘Antibiotic resistance’ was among the highest priorities (scale 1–10) with an average of 8.86 (SEM ± 0.05), together with ‘Gender inequality’ (8.68 ± 0.07) and ‘Climate change’ (8.68 ± 0.07). Only 28.8% thought that Dentistry was engaged in national/international campaigns promoting awareness on the topic and 8.9% stated to have heard about the ‘One Health’ concept. Final year dental students showed an average confidence to prescribe antibiotics of 7.59 (± 0.14). Most students demonstrated interest in receiving additional education on all topics listed, with the three most pressing being ‘antibiotic prescription for treatment of infections’ (82.9%), ‘drug interactions’ (80.9%), and ‘spread of antibiotic resistance’ (79.6%). A trend was observed between higher awareness regarding the topic and higher confidence to prescribe.

**Conclusions:**

There is a need to revisit dental education on antibiotic resistance with a global perspective and to create more stewardship initiatives that promote awareness on the topic.

**Supplementary Information:**

The online version contains supplementary material available at 10.1186/s13756-022-01192-x.

## Introduction

The antimicrobial resistance (AMR) crisis is one of the biggest threats to global health, food security, and societal development [1]. Not only a growing number of infections are becoming more difficult to treat as pathogenic microbes develop or acquire resistance to antibiotics, but also several modern medical treatments such as Caesarian sections, organ transplantations, immunosuppressive chemotherapy, and implant insertions are at risk since they depend on the availability of effective antibiotic therapy [1, 2]. Although antibiotic resistance is a phenomenon that occurs naturally, the overuse and misuse of antibiotics in humans and animals is a major driver of this process [4, 5]. As such, optimization of antimicrobial use in humans and animals has been one of the five main objectives in the ‘Global Action Plan on Antimicrobial Resistance’ published by the World Health Organization (WHO) in 2015 [6]. This remains a high priority in the healthcare agenda as highlighted by the ‘No Time to Wait: Securing the future from drug-resistant infections’ report to the Secretary-general of the United Nations (UN) in 2019 [7] and by UN Cooperation Framework on AMR for sustainable development goals [8]. In dentistry, a recent white paper by the FDI Word Dental Federation addresses the role of dental teams in the fight against the AMR crisis [10].

The majority of human antibiotic consumption takes place in primary care [4, 11, 12], which includes prescriptions in oral health care. In general, it is reported that dentists prescribe about 10% of antibiotics for humans [10], however, such rate can vary depending on the country. For instance, in Norway, around 85% of the human use of antibacterial drugs is employed in ambulatory care, and dentists are responsible for 5.9% of these antibiotic prescriptions [11]. The latest report by the English surveillance programme for antimicrobial utilisation and resistance (ESPAUR) indicates that antibiotics prescribed within the general practitioner setting in 2020 accounted for 72.7% of all prescriptions, with 4.7% attributed to dentists [12]. In the United States, data indicate that dentists are responsible for 10% of antibiotic prescriptions [13] and are one of the top prescribers when grouped by specialty area [14]. It is important to note, however, that there is limited or no available information on prescription rates by dentists in a variety of world regions..Recent studies analyzing prescription patterns show a high number of unnecessary prescriptions for both prophylaxis and treatment in dentistry. In a retrospective cohort study in the US, Suda and collaborators investigated the appropriateness of antibiotic prescriptions for dental prophylaxis by looking at retrospective insurance data and reported that more than 80% of the prescriptions were considered unnecessary [15]. In the province of British Columbia in Canada, from 1996 to 2013, while overall prescription of antibiotics declined 12.8%, dental prescribing increased 62.2% [16]. In Norway, a survey from 2017 among dental practitioners indicated several areas of knowledge with room for improvement [17]. Antibiotic prescriptions by dentists have been decreasing in this country over recent years, and the overall number of prescriptions is considered to be low [11]. In India, a recent systematic review identified significant misuse of antibiotics in dentistry, both for treatment and prophylaxis [18]. Multiple studies with different populations indicate that dental prescribing practices can be vastly improved [19–22]. Further, a recent population-level analysis indicated concerning differences in the patterns of antibiotic prescriptions among dentists worldwide [23].

Here we assessed awareness, perceptions, and confidence to prescribe antibiotics among dental students from six universities in Norway, Canada, and Brazil. In particular, we aimed to (i) evaluate the students’ perceptions and awareness regarding the antibiotic resistance crisis; (ii) understand the students’ level of confidence regarding antibiotic prescribing practices in various scenarios; (iii) reveal if students perceived a need for more education on topics regarding antibiotic resistance.

## Materials and methods

### Study design and population

We conducted a cross-sectional survey among dental students from six universities located in Canada (University of Toronto and McGill University), Brazil (University of Campinas and Federal University of Rio Grande do Sul), and Norway (University of Oslo and University of Bergen). All students enrolled in the Faculty of Dentistry at these universities were contacted by e-mail with a link to the questionnaire survey hosted in a secure server in Norway (Nettskjema). In addition to sending up to three reminder e-mails, students were encouraged to participate in the survey by lecturers/instructors and closed social media groups. Participation was anonymous, voluntary, and no compensation was offered. While all dental students were invited to answer the first section of the questionnaire, only final-year dental students answered the second and third sections.

### Questionnaire design

The questionnaire (Additional file 1: Fig. S1) was constructed by a multidisciplinary group of professionals involved with teaching and research at higher-education institutions and governmental agencies. The suitability of the instrument was ensured through content validity, face validity, and a pilot test with a convenience sample. The initial step of the construction was the definition of the domain and scope of the questionnaire. This stage of content validity included the formulation of relevant questions by the research team coupled with a thorough literature search and the adaptation of items used in previous survey instruments [24–26]. For face validity, a panel of experts composed of two microbiologists, two epidemiologists, and two clinicians were invited to evaluate the instrument and their concerns were addressed. The final questionnaire contained fourteen questions in total and was divided in three different sections: (i) Perceptions and knowledge, comprising six questions; (ii) Experiences and confidence, with four questions; and (iii) Improvement and continuing education, containing four items. Close-end and multiple-choice questions comprised the core of the instrument as they provide structure and avoid fatigue by the respondents. In the last question of the instrument, however, an open-ended question was available for participants to express their opinions that had not been addressed in the previous items. For reliability, the instrument was translated and back-translated from English to Portuguese and Norwegian by native experts. The final construct was pre-tested in a convenience sample of ten participants that contained native speakers of each of the three languages encompassed in the questionnaire.

### Statistical analyses

Descriptive analyses were carried out with Excel from the Microsoft Office package (Redmond, WA, USA) and IBM SPSS 28.0 (Armonk, NY, USA). At times, groups of answers were merged to facilitate interpretation. Metric variables were reported as means with standard error of the mean (± SEM) and medians. Tests for comparison between two groups were performed with Mann–Whitney test, and for comparison between three or more groups, Kruskal–Wallis test was employed for nonparametric data. Further, chi-square was employed for comparison of discrete data. The level of significance was set at α = 0.05 (two‐sided). For data visualization, graphs were prepared with the software GraphPad Prism 9 (San Diego, CA, USA).

### Ethical considerations

This study was conducted in accordance with the Declaration of Helsinki and national and institutional standards. Participation in the survey was voluntary and anonymous, and only sex and age were collected for personal data. All records were directly stored in the secure servers of https://nettskjema.no/, thus minimizing the risk of adverse events connected to the utilization of digital vehicles for research such as viruses and malware. The project obtained clearance from the Ethics Research Committee in Norway and was approved by the Norwegian Centre for Research Data (no. 285858). Further, Institutional Review Board approval was obtained in Canada (no. A06-B43-20A (20–06-015)) and Brazil (no. 4.255.234). Participation consent was presented and collected in the initial page of the online questionnaire.

## Results

### Response rate and demographics

In total, 562 students from the six universities in Norway, Canada, and Brazil completed the questionnaire. Response rates averaged 28.6% varying between universities in Norway (33.5%, n = 172), universities in Canada (25.4%, n = 150), and universities in Brazil (30%, n = 240). Overall, the distribution of sociodemographic factors was similar among the students, with exception of Canada that presented a higher percentage of males (Table 1). In Brazil, it was observed a higher percentage of student involvement in research activities (44.6%) followed by Canada (24.7%) and Norway (8.1%) (Table 1).

**Table 1:** Characteristics of the study population

	Norway	Canada	Brazil	Total
*Total participants*				
(n, %)	172 (100)	150 (100)	240 (100)	562 (100)
*Age*				
Mean (SEM, Median)	24.16 (± 0.36, 23)	25.62 (± 0.32, 25)	22.21 (± 0.25, 22)	23.72 (± 0.19, 23)
*Sex*				
Female (n, %)	129 (75)	91 (60.7)	182 (75.8)	402 (71.5)
Male (n, %)	43 (25)	59 (39.3)	58 (24.2)	160 (28.5)
*Research activities*				
(n, %)	14 (8.1)	37 (24.7)	107 (44.6)	158 (28.1)
*Final year students*				
(n, %)	41 (23.8)	39 (26)	72 (30)	152 (27)
*Interest in work placement for final year students (n, %)*				
Private sector	25 (61)	35 (89.7)	52 (72.2)	112 (73.7)
Public sector	28 (68.3)	15 (38.5)	43 (59.7)	86 (56.6)
Academia	3 (7.3)	10 (25.6)	12 (16.7)	25 (16.4)
Other	1 (2.4)	2 (5.1)	0 (0)	3 (2)
Unsure	3 (7.3)	2 (5.1)	6 (8.3)	11 (7.2)

### Perceptions and knowledge

**Fig. 1:**
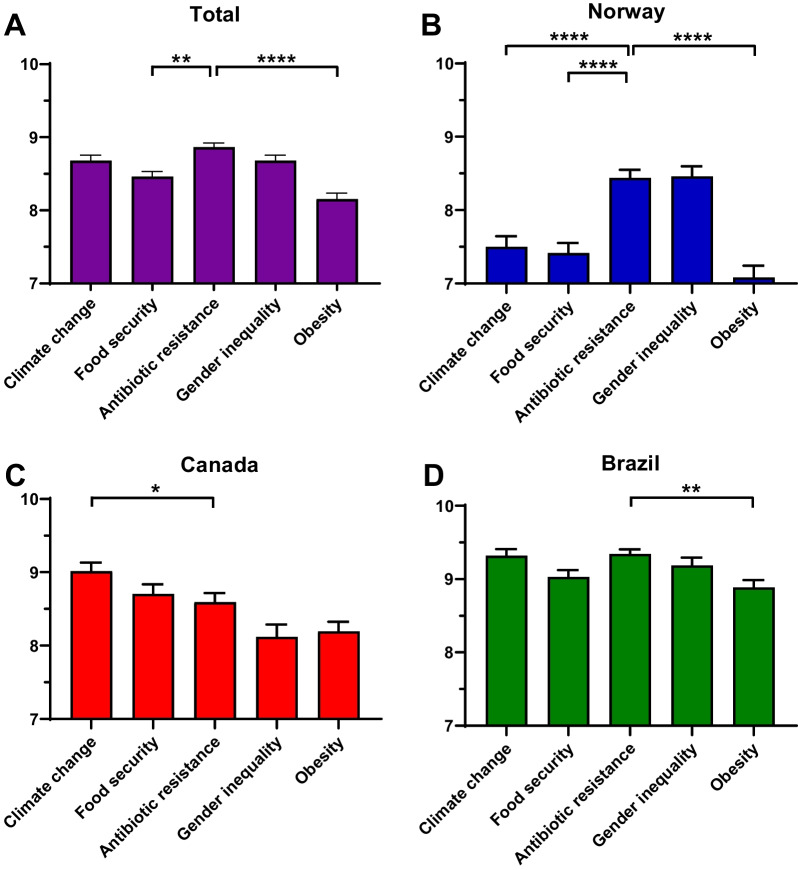
Perceived awareness on global issues for dental students on a scale from 1 to 10. **A** All participants in the survey, **B** participants from Norway, **C** participants from Canada, and **D** participants from Brazil. Statistics were performed with Kruskal–Wallis one-way analysis of variance with Dunn’s posthoc with the primary basis of comparison being ‘Antibiotic resistance’. **p < 0.01, ****p < 0.0001

**Fig. 2:**
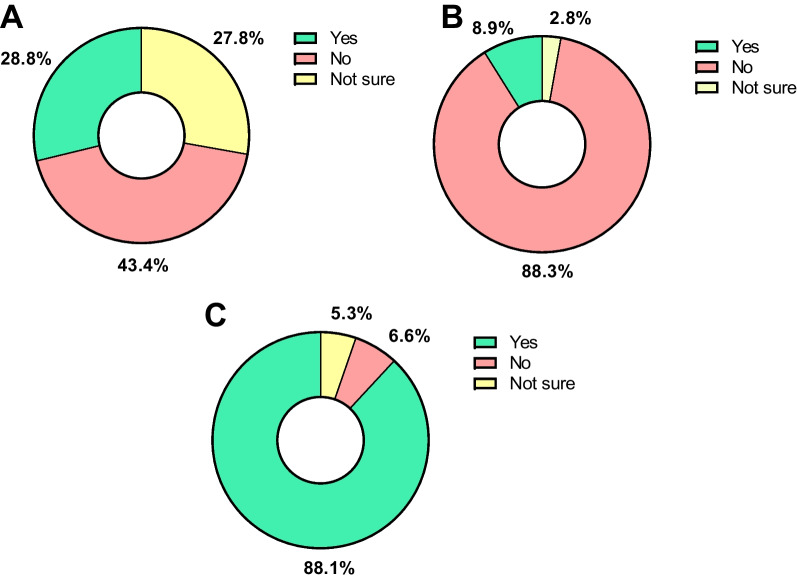
Knowledge of students regarding the involvement of dentistry in the antibiotic resistance crisis response. **A** Engagement in national and international campaigns promoting awareness on antibiotic resistance. **B** Knowledge of the concept of One Health. **C** Importance of the topic of antibiotic resistance for dentists

Overall, among several global challenges, ‘antibiotic resistance’ was placed at the highest priority level with an average of 8.86 (± 0.05), together with ‘gender inequality’ (8.68 ± 0.07) and ‘climate change’ (8.68 ± 0.07) (Fig. 1A). Nevertheless, the patterns changed when we analyzed the data by country (Fig. 1B–D). Only 28.8% of dental students thought that dentistry was engaged in national and international campaigns that promote awareness on antibiotic resistance (Fig. 2). In total, 88.3% had never heard about ‘One Health’ (Fig. 2B). Further, the majority of students stated that antibiotic resistance is an important topic for dentists (Fig. 2C). From the 6.6% (n = 37) that replied negatively, 91.9% were not in the final year of their studies.

**Fig. 3:**
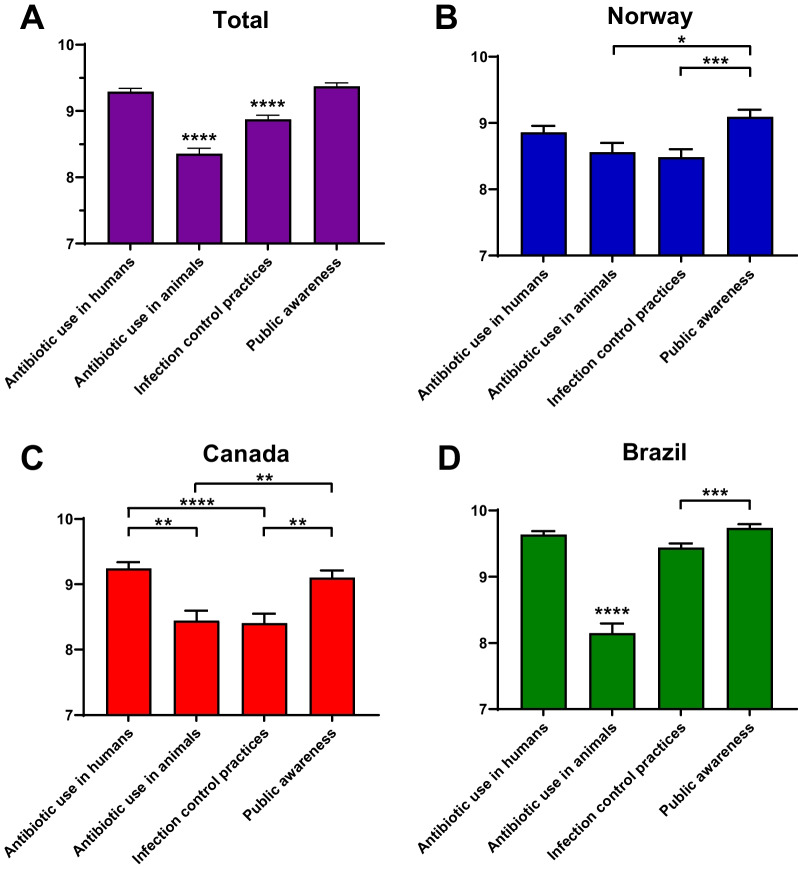
Perception of dental students on which areas should be addressed to slow down the development of antibiotic resistance on a scale from 1 to 10. **A** All participants in the survey, **B** participants from Norway, **C** participants from Canada, and **D** participants from Brazil. Statistics were performed with Kruskal–Wallis one-way analysis of variance with Dunn’s posthoc. ****p < 0.0001

The ‘inappropriate use of antibiotics in humans’ and ‘general public awareness on antibiotic resistance’ were considered the two main areas to focus on in the fight against antibiotic resistance (Fig. 3A). The same finding was observed for students in each country individually (Fig. 3B–D). When considering all participants in the survey, these two topics were placed significantly higher (*p* < 0.0001) than the other options. Additional file 1: Table S1 shows absolute and relative values for dental students’ agreement level regarding a variety of statements on antibiotic resistance.

### Prior experience and confidence to prescribe

**Fig. 4:**
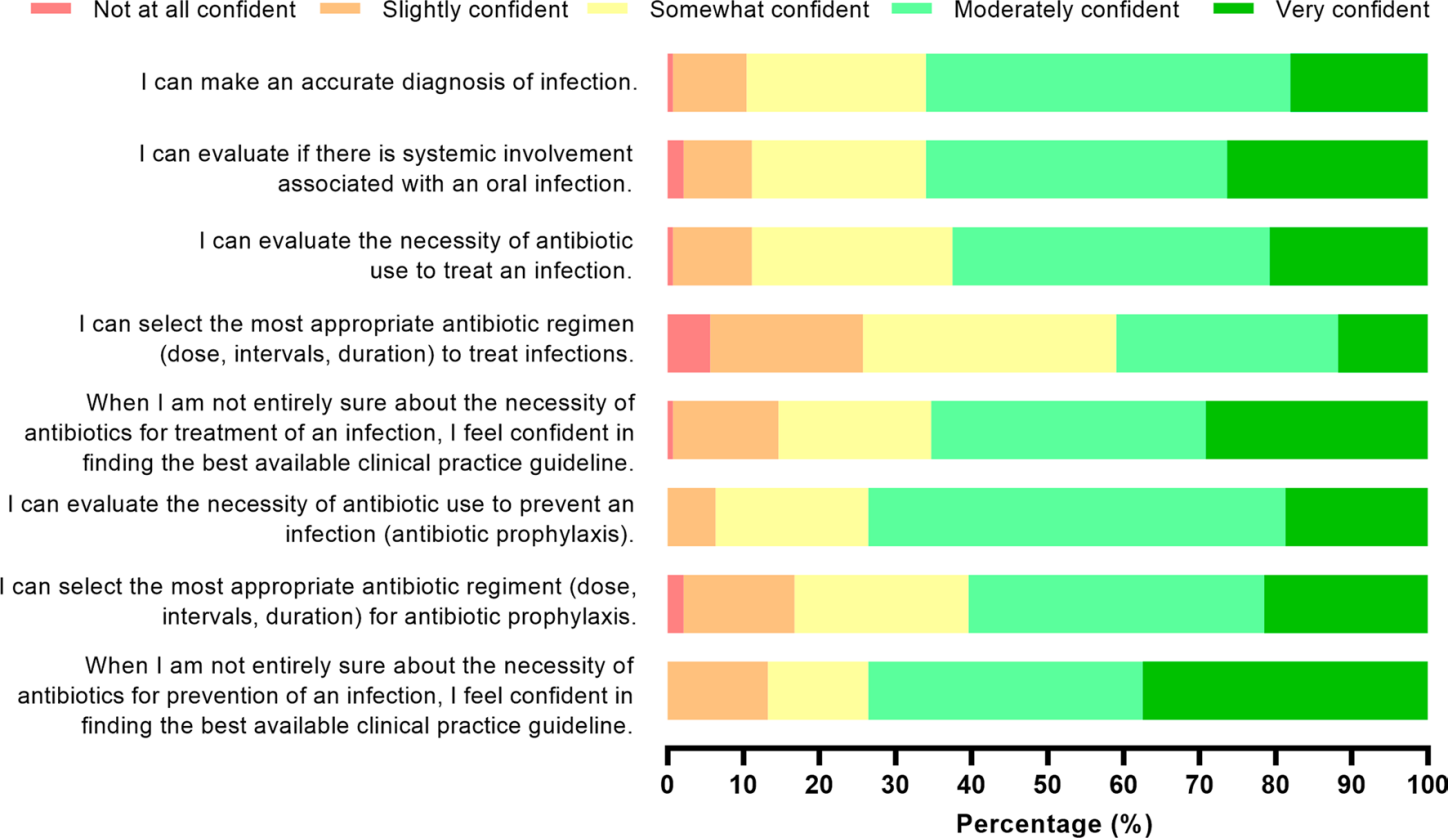
Participants’ level of confidence concerning antibiotic prescriptions for treatment (five first statements) and prevention of infections (three last statements). Relative values (%) of responses are represented in the x-axis

**Fig. 5:**
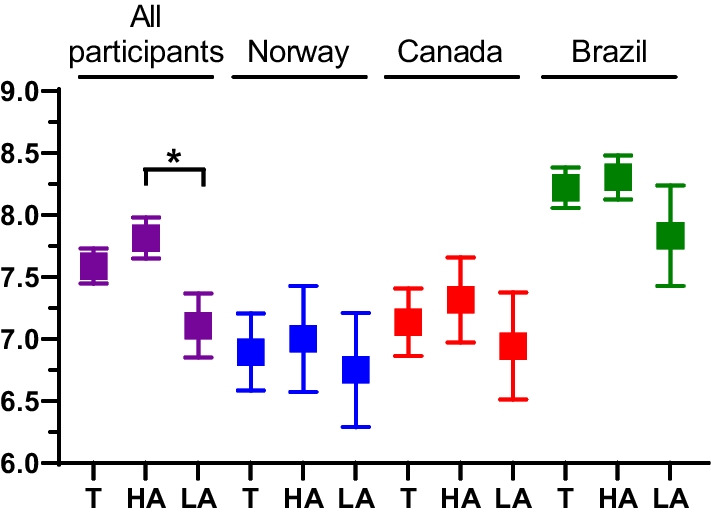
Level of confidence of final year dental students to prescribe antibiotics on a scale from 1 (I do not have enough knowledge) to 10 (I have enough knowledge). Symbols show averages of answers with error bars representing standard error of the mean (SEM). ‘T’ indicates total responses; ‘HA’—higher awareness on antibiotic resistance; ‘LA’—lower awareness on antibiotic resistance. Statistics comparing confidence level between higher and lower awareness groups were performed with Mann–Whitney test. *p < 0.05

The majority of final-year students answered that they were moderately confident or very confident to prescribe antibiotics in different clinical scenarios provided (Fig. 4), with the exception being the selection of antibiotic regimen to treat infections, in which students showed less confidence. Average confidence to prescribe was 7.59 (± 0.14) across all countries (Fig. 5). For comparison analysis with awareness values, students were divided into ‘higher awareness’ (n = 103 that marked 9 or 10 in the scale from the previous section) and ‘lower awareness’ (n = 49 that marked 8 or lower). As such, the trend that students with higher awareness also presented more confidence concerning antibiotic prescription was observed across all countries (Fig. 5). Concerning confidence to communicate to patients when antibiotics are not needed, 25.7% stated ‘Very confident’, 43.1% ‘Moderately confident’, 16.7% ‘Somewhat confident’, 7.6% ‘Slightly confident’, and 6.9% ‘Not at all confident’. Further, almost half of the population (44.7%) stated to rarely speak about antibiotic resistance with their patients (Additional file 1: Fig. S2). Although not statistically significant, more students in the ‘lower awareness’ group expressed that they never talk about antibiotic resistance with their patients (14/49, 28.6%) compared to the ‘higher awareness’ group (16/103, 15.5%).

### Further education

**Fig. 6:**
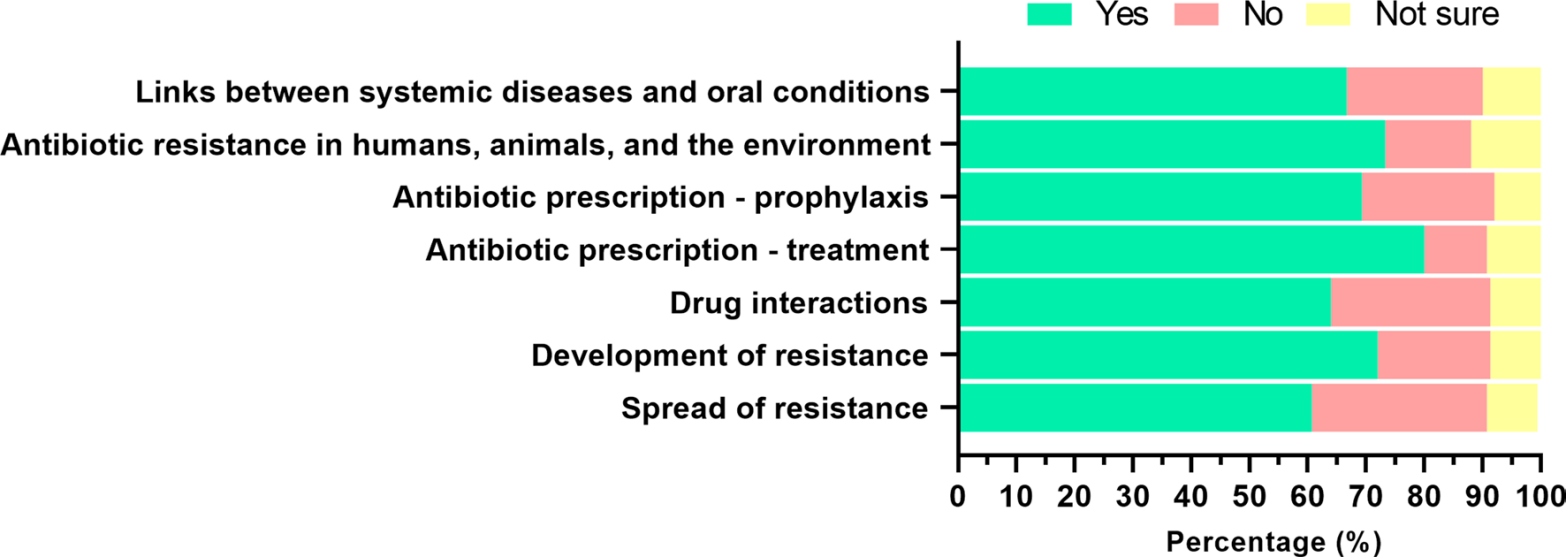
Interest of final year dental students in receiving further education and information on selected topics related to antibiotic resistance. Relative values (%) of responses are represented in the x-axis

A large number of final-year students reported interest for further education in ‘antibiotic prescription for treatment of infections’ (82.9%), ‘drug interactions’ (80.9%), and ‘spread of antibiotic resistance’ (79.6%) (Fig. 6). In terms of how to address these topics, preferred types of education (selected as ‘useful’ or ‘very useful’) were ‘teaching in small groups’ (78.2%), ‘lectures at the university’ (73.7%), and ‘online courses’ (59.9%). Most students (67.8%) responded positively to the added benefit of having focused material such as pamphlets to engage in conversations about antibiotic resistance with patients.

## Discussion

This study investigated the awareness, perceptions, and experience of dental students in three different countries and continents regarding antibiotic resistance. Results indicate that, despite some differences across countries, dental students as a whole identify ‘Antibiotic resistance’ as the main challenge compared to the other topics raised in the questionnaire. However, general integration of dentistry with other areas of human and environmental health seemed poor considering that only 8.9% reported to have heard about the ‘One Health’ concept and less than a third thought that dentistry was engaged in campaigns against antibiotic resistance. It was also observed that confidence to prescribe should be improved and there was a same direction trend between higher awareness on the topic and higher confidence to prescribe. Finally, dental students presented a strong interest in receiving additional education on a variety of different topics concerning antibiotic resistance. Our data support the rationale that programs promoting proper and evidence-based prescription practices, *i.e.* antimicrobial stewardship, in dentistry may be vital instruments in preparing dental students to prescribe antibiotics consciously and act as disseminators of knowledge to their patients and the public [27].

Awareness of the challenge of antibiotic resistance is a key element when addressing initiatives that raise attention on proper antibiotic prescribing practices and can often facilitate communication, understanding, and acceptance between peers and the public [27, 28]. When asked to grade five different proposed challenges, dental students rated ‘Antibiotic resistance’ as one of the highest together with ‘Climate change’ and ‘Gender inequality’. A recent study in the United Kingdom evaluated the awareness of university students from 25 institutions across a variety of courses, including dentistry. There were 255 students enrolled and, from those, 11 were dental students [25]. As a whole, the topic of ‘antibiotic resistance’ (mean of 9.0) was graded above all other four—climate change (8.4), food security (7.7), gender inequality (7.3), and obesity (8.0). In our study, ‘antibiotic resistance’ was graded as 8.86, which remains similar to the results presented from the UK, despite expected divergences from being a different population. Most students indicated that antibiotic resistance is an important topic for dentists, and the few that responded negatively were students not in the final year of their studies. This suggests a positive effect of increasing awareness during the course and highlights the importance to emphasize the topic also early in the studies.

The ‘One Health’ approach aims to design and implement programs, research, policies, and legislation anchored on the communication and interplay between multiple sectors to achieve better public health outcomes. Such strategy is particularly relevant in light of the AMR crisis (for review see [29]). Despite the awareness presented by dental students in this study, surprisingly few students had heard of ‘One health’ and only a third of students thought that dentistry was engaged in national and international campaigns to fight antibiotic resistance. This is concerning as the integration of dentistry in a collaborative, multisectoral, and transdisciplinary approach is central to ensure proper oral health care to global populations. In health sciences, the issue of ‘One health’ integration has been suggested previously [30–32], and interventions that allow for the adaptation of the area into the curricula [33, 34] are warranted in dentistry. Such need is also corroborated in this study by the fact that students perceived the ‘inappropriate use of antibiotics in animals’ as significantly less relevant in terms of hindering the development of antibiotic resistance when compared to the other suggestions.

Confidence to prescribe antibiotics is an important element of dental education. While lack of confidence does not necessarily translate to poor practice, it may act as a stressor, which can hinder clinical decision-making and can affect negatively the practice’s routines [35]. Final year dental students in this study showed an average confidence of 7.59 from 1 to 10. In the specific scenarios, the students felt the least confident to select the most appropriate antibiotic and regimen to treat infections, which is consistent with the findings of other studies both in the UK and the US [25, 36]. This also goes in line with previous studies assessing health care students’ confidence and preparedness to prescribe [25, 37, 38]. The present study adds to the body of knowledge regarding antibiotic use and can act as a basis for continuing studies monitoring confidence to prescribe by dental students. In addition, further studies should focus on aspects that evaluate the appropriateness to prescribe by using different tools such as case vignettes and include multivariate analyses to explore determinants that affect confidence to prescribe in a representative population of dental students and dentists.

The data in this study strongly indicate the interest of final year dental students across the three countries to receive more education and information on the topics concerning antibiotic resistance. These topics span across basic sciences with areas such as the development and spread of resistance, clinical sciences when addressing antibiotic prescription practices and drug interactions, and public health with the links between antibiotic resistance in humans, animals and the environment. Altogether, such findings suggest that more attention towards ‘antibiotic resistance’ is necessary in different areas of the curriculum. An integrative approach would contribute in highlighting the importance of this global health challenge [10]. A recent study from Poland investigated final-year dental students’ knowledge and practices regarding antibiotic prescription with the use of a self-administered questionnaire. The authors found largely divergent levels of awareness and behavior, which emphasized the need to educate dental students on antibiotic use and its inherent risks [39]. Another study within US dental schools identified the need to revise dental curricula regarding the appropriate use of antibiotics [36]. Such findings are not exclusive to dentistry and have also been identified in a number of studies investigating the knowledge and practices of medical students with regards to antibiotic prescription practices [24, 37, 38, 40]. As prescribers need to ensure the optimal outcome for each patient but also for the long-term of public health, decision-making on antibiotic use is a challenging step, particularly in light of the antibiotic resistance crisis [41]. However, facilitating healthy habits as early as possible while students are shaping their behavior is a relevant strategy to promote prudent and evidence-based antimicrobial prescription practices [42, 43]. Further, the implementation of online courses as a supplement to dental undergraduate education can increase student understanding of antibiotic resistance and antimicrobial stewardship [44]. It can also be hypothesized that such tools would be helpful as a refresher course for trained dentists.

The initial design of this study was to administer the questionnaire in person to each group of students just before dental classes in the fall semester of 2020. However, due to the unforeseen situation with the COVID-19 pandemic, the strategy was shifted to an online survey. As such, students were invited by e-mail and sent up to three reminders. On average, a response rate of ~ 30% was obtained and this goes in line with previous online surveys on the topic [24, 38, 45, 46]. Furthermore, it is not believed that a significant selection bias has been introduced given the topic of the study and responses obtained are highly relevant to the educational systems involved and the field of dentistry. Regardless, caution should be exercised when interpreting the results. The questionnaire was anonymous and it was made clear to students that no identifiable variables were utilized. However, one should consider the possibility of the Hawthorne effect, which can be defined as a form of reactivity where subjects modify their behavior in response to being observed [47].

## Conclusions

In this study, it was shown that despite presenting a seemingly high awareness towards antibiotic resistance, students lack in comprehension regarding how human health and the environment are interlinked. The indication that higher awareness is connected to more confidence to prescribe is promising and supports the creation and maintenance of stewardship programs aiming to address antibiotic resistance in dental schools. In addition, the study revealed areas for improvement regarding confidence to prescribe and, in general, dental students perceived a large need to better address areas of importance regarding antibiotic resistance education throughout the dental curriculum.

## Supplementary Information

**Additional file 1.** Supplementary material to the paper Awareness regarding antimicrobial resistance and confidence to prescribe antibiotics in Dentistry: a cross-continental student survey.

## Data Availability

The datasets used and/or analyzed during the current study are available from the corresponding author on reasonable request.
