# Supplementary material for: Awareness regarding antimicrobial resistance and confidence to prescribe antibiotics in dentistry: a cross-continental student survey
Source: Antimicrob Resist Infect Control. 2022 Dec 11;11:158. doi: 10.1186/s13756-022-01192-x (PMC9741920; doi:10.1186/s13756-022-01192-x)
Supplement: Supplementary file 1 — Additional file 1. Supplementary material to the paper Awareness regarding antimicrobial resistance and confidence to prescribe antibiotics in Dentistry: a cross-continental student survey. [file 13756_2022_1192_MOESM1_ESM.pdf]

Additional file 1. Supplementary material to the paper *Awareness regarding antimicrobial resistance and confidence to prescribe antibiotics in Dentistry: a cross-continental student survey*.

Additional file 1: Fig. S1. Questionnaire used in the study.

Awareness regarding antimicrobial resistance and confidence to prescribe antibiotics in Dentistry: a cross-continental student survey

Sex: ☐ Female ☐ Male Age: ____ years

Do you participate in research activities? If yes, in which area? ☐ No ☐ Yes: _____

Where do you see yourself working in the future? ☐ Private sector ☐ Public sector ☐ Other ☐ Academia ☐ I don't know

Are you in the final year of your studies?

☐ Yes, I am in the final year of my studies.

☐ No, I am NOT in the final year of my studies.

In which year are you expected to graduate? ____________

PART I: Perceptions and knowledge

1) How important do you think the following challenges are in the world today? Scale from 1 (not at all important) to 10 (extremely important):

	1	2	3	4	5	6	7	8	9	10	I don't know
Climate change	☐	☐	☐	☐	☐	☐	☐	☐	☐	☐	☐
Food security	☐	☐	☐	☐	☐	☐	☐	☐	☐	☐	☐
Antibiotic resistance	☐	☐	☐	☐	☐	☐	☐	☐	☐	☐	☐
Gender inequality	☐	☐	☐	☐	☐	☐	☐	☐	☐	☐	☐
Obesity	☐	☐	☐	☐	☐	☐	☐	☐	☐	☐	☐

2) To your knowledge, is Dentistry engaged in participating in national and international campaigns that promote awareness on antibiotic resistance?

Yes ☐ No ☐ I don't know ☐

3) Have you heard about 'One Health'?

Yes ☐ No ☐ I don't know ☐

4) Do you think that 'antibiotic resistance' is an important topic for dentists?

Yes ☐ No ☐ I don't know ☐

5) Please select the option that best suits your level of agreement with each statement. Scale from 'Strongly disagree' to 'Strongly agree':

	Strongly disagree	Disagree	Somewhat disagree	Neutral	Somewhat agree	Agree	Strongly agree
The overuse of antibiotics is a driver of antibiotic resistance.	☐	☐	☐	☐	☐	☐	☐
The overuse of antibiotics prescribed by dentists contributes to antibiotic resistance.	☐	☐	☐	☐	☐	☐	☐
The number of newly discovered antibiotics has increased in recent years.	☐	☐	☐	☐	☐	☐	☐
Vaccination and personal hygiene are important to reduce the number of antibiotics used in the world.	☐	☐	☐	☐	☐	☐	☐
Self-medication with antibiotics contributes to the increase of antibiotic resistance.	☐	☐	☐	☐	☐	☐	☐
Antibiotic resistance is a significant threat in the country where I live.	☐	☐	☐	☐	☐	☐	☐
To prescribe unnecessary antibiotics is unethical.	☐	☐	☐	☐	☐	☐	☐
The phenomenon of antibiotic resistance is mainly a problem in hospital settings.	☐	☐	☐	☐	☐	☐	☐
I understand how antibiotic resistance is developed.	☐	☐	☐	☐	☐	☐	☐
I understand how antibiotic resistance is spread.	☐	☐	☐	☐	☐	☐	☐
I think the problem of antibiotic resistance will increase over the next years.	☐	☐	☐	☐	☐	☐	☐
I am convinced that new antibiotics will be developed to solve the problem of resistance.	☐	☐	☐	☐	☐	☐	☐

6) In your opinion, which areas should be targeted to slow down the development of antibiotic resistance? Scale from 1 (not at all important) to 10 (extremely important):

	1	2	3	4	5	6	7	8	9	10	I don't know
Inappropriate use of antibiotics in humans.	☐	☐	☐	☐	☐	☐	☐	☐	☐	☐	☐
Inappropriate use of antibiotics in animals.	☐	☐	☐	☐	☐	☐	☐	☐	☐	☐	☐
Hygiene and infection control practices.	☐	☐	☐	☐	☐	☐	☐	☐	☐	☐	☐
General public awareness on antibiotic resistance.	☐	☐	☐	☐	☐	☐	☐	☐	☐	☐	☐

PART II: Experiences and confidence *(Final-year students)*

7) Do you feel you have sufficient knowledge about antibiotic use for your clinical practice? Scale from 1 (I do not have enough knowledge) to 10 (I have enough knowledge):

1	2	3	4	5	6	7	8	9	10	I don't know
☐	☐	☐	☐	☐	☐	☐	☐	☐	☐	☐

8) How confident do you feel to communicate to patients when antibiotics are not needed?

Not at all confident	Slightly confident	Somewhat confident	Moderately confident	Very confident
☐	☐	☐	☐	☐

9) Please select the option that best suits your level of agreement with each statement. Scale from 'Strongly disagree' to 'Strongly agree':

	Strongly disagree	Disagree	Somewhat disagree	Neutral	Somewhat agree	Agree	Strongly agree
When there is lack of time, I feel more pressured to prescribe antibiotics.	☐	☐	☐	☐	☐	☐	☐
In certain situations, I feel pressured by patients to prescribe antibiotics.	☐	☐	☐	☐	☐	☐	☐

10) Please select the option that best suits your level of confidence concerning each statement about prescription of antibiotics for treatment and prevention of infections of oral origin in Dentistry:

	Not at all confident	Slightly confident	Somewahť confident	Moderately confident	Very confident
**Treatment of infections with antibiotics**					
I can make an accurate diagnosis of infection.	☐	☐	☐	☐	☐
I can evaluate if there is systemic involvement associated with an oral infection.	☐	☐	☐	☐	☐
I can evaluate the necessity of antibiotic use to treat an infection.	☐	☐	☐	☐	☐
I can select the most appropriate antibiotic and regimen (dose, intervals, duration) to treat infections.	☐	☐	☐	☐	☐
When I am not entirely sure about the necessity of antibiotics for treatment of an infection, I feel confident in finding the best available clinical practice guideline.	☐	☐	☐	☐	☐
**Prevention of infections with antibiotics (prophylaxis)**					
I can evaluate the necessity of antibiotic use to prevent an infection (antibiotic prophylaxis).	☐	☐	☐	☐	☐
I can select the most appropriate antibiotic and regimen (dose, intervals, duration) for prevention of infections (antibiotic prophylaxis).	☐	☐	☐	☐	☐
When I am not entirely sure about the necessity of antibiotics for prevention of an infection (antibiotic prophylaxis), I feel confident in finding the best available clinical practice guideline.	☐	☐	☐	☐	☐

PART III: Education *(Final-year students)*

11) Please select if you think you should receive more education/information about the topics below as part of your education.

	Yes	No	I don't know
Development of antibiotic resistance.	☐	☐	☐
Spread of antibiotic resistance.	☐	☐	☐
Drug interactions.	☐	☐	☐
Antibiotic prescription for treatment of infections.	☐	☐	☐
Antibiotic prescription for prevention of infections (antibiotic prophylaxis).	☐	☐	☐
Antibiotic resistance in humans, animals, and the environment.	☐	☐	☐
Links between systemic diseases and oral conditions.	☐	☐	☐

12) From the following options for further education, please select which you would prefer considering antibiotic resistance and prescription practices:

	Not useful	Neutral	Useful	Very useful
Lectures (>15 participants)	☐	☐	☐	☐
Teaching in small groups (<15 participants)	☐	☐	☐	☐
Online courses	☐	☐	☐	☐
Pamphlets	☐	☐	☐	☐
Newsletters	☐	☐	☐	☐
Online discussion groups	☐	☐	☐	☐

13) How often do you talk about antibiotic resistance with your patients?

Never ☐ Rarely ☐ Sometimes ☐ Often ☐ Very often ☐

14) Would having additional material (such as informative pamphlets) be useful to engage patients regarding prudent antibiotic use?

Yes ☐ No ☐ I don't know ☐

15) If you would like, please use this section to provide additional comments or questions.

Thank you very much for completing the survey!

Additional file 1: Table S1. Level of agreement of dental students in relation to twelve statements on antibiotic resistance. Results are presented both in absolute and relative values. For visualization, darker shades of orange represent higher frequency of responses

	Strongly disagree n (%)	Disagree n (%)	Somewhat disagree n (%)	Neutral n (%)	Somewhat agree n (%)	Agree n (%)	Strongly agree n (%)
The overuse of antibiotics is a driver of antibiotic resistance.	3 (0.5)	1 (0.2)	3 (0.5)	1 (0.4)	28 (5.0)	169 (30.1)	356 (63.3)
The overuse of antibiotics prescribed by dentists contributes to antibiotic resistance.	4 (0.7)	9 (1.6)	25 (4.4)	63 (11.2)	99 (17.6)	176 (31.3)	186 (33.1)
The number of newly discovered antibiotics has increased in recent years.	60 (10.7)	108 (19.2)	39 (6.9)	229 (40.7)	62 (11.0)	53 (9.4)	11 (2.0)
Vaccination and personal hygiene are important to reduce the number of antibiotics used in the world.	2 (0.4)	11 (2.0)	20 (3.6)	52 (9.3)	103 (18.3)	209 (37.2)	165 (29.4)
Self-medication with antibiotics contributes to the increase of antibiotic resistance.	2 (0.4)	4 (0.7)	5 (0.9)	8 (1.4)	37 (6.6)	164 (29.2)	342 (60.9)
Antibiotic resistance is a significant threat in my country of residence.	1 (0.2)	10 (1.8)	18 (3.2)	93 (16.5)	103 (18.3)	221 (39.3)	116 (20.6)

To prescribe unnecessary antibiotics is unethical.	4 (0.7)	3 (0.5)	10 (1.8)	27 (4.8)	73 (13.0)	155 (27.6)	290 (51.6)
The phenomenon of antibiotic resistance is mainly a problem in hospital settings.	70 (12.5)	147 (26.2)	69 (12.3)	78 (13.9)	85 (15.1)	69 (12.3)	44 (7.8)
I understand how antibiotic resistance is developed.	3 (0.5)	18 (3.2)	18 (3.2)	43 (7.7)	152 (27.0)	229 (40.7)	99 (17.6)
I understand how antibiotic resistance is spread.	0 (0.0)	16 (2.8)	24 (4.3)	47 (8.4)	152 (27.0)	227 (40.4)	96 (17.1)
I think the problem of antibiotic resistance will increase over the next years.	1 (0.2)	0 (0.0)	5 (0.9)	30 (5.3)	73 (13.0)	232 (41.3)	221 (39.3)
I am convinced that new antibiotics will be developed to solve the problem of resistance.	24 (4.3)	125 (22.2)	137 (24.4)	133 (23.7)	81 (14.4)	50 (8.9)	12 (2.1)

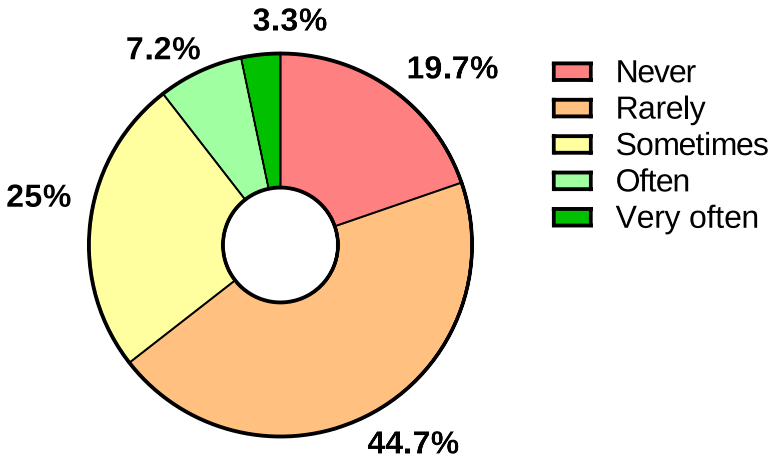

Additional file 1: Fig. S2. Frequency of communication about antibiotic resistance between dental students and their patients.
